# A phase II study to evaluate the safety and efficacy of anlotinib combined with toripalimab for advanced biliary tract cancer

**DOI:** 10.1002/cti2.1483

**Published:** 2024-01-12

**Authors:** Mingzhen Zhou, Yuncheng Jin, Sihui Zhu, Chen Xu, Lin Li, Baorui Liu, Jie Shen

**Affiliations:** ^1^ Comprehensive Cancer Centre, Department of Oncology, Nanjing Drum Tower Hospital The Affiliated Hospital of Nanjing University Medical School Nanjing China; ^2^ Department of Oncology, Nanjing Drum Tower Hospital, Clinical College of Traditional Chinese and Western Medicine Nanjing University of Chinese Medicine Nanjing China; ^3^ International Hospital Affiliated to Medical School of Nanjing University Nanjing China; ^4^ Department of Pathology, Nanjing Drum Tower Hospital The Affiliated Hospital of Nanjing University Medical School Nanjing China; ^5^ Department of Precision Medicine, Nanjing Drum Tower Hospital The Affiliated Hospital of Nanjing University Medical School Nanjing China

**Keywords:** biliary tract cancer, biomarkers, immunotherapy, targeted therapy

## Abstract

**Objectives:**

To assess the safety and efficacy of anlotinib (a multi‐targeted tyrosine kinase inhibitor) combined with toripalimab (a PD‐1 monoclonal antibody) in the treatment of unresectable biliary tract cancer (BTC).

**Methods:**

In this prospective, single‐arm, single‐centre exploratory clinical study, patients with locally progressed or metastatic BTC were included. Patients were treated with anlotinib (12 mg, PO, QD, for 2 weeks and then stopped for a week, 21 days for a cycle) and toripalimab (240 mg, IV, Q3W). The primary endpoint of the study was the objective response rate (ORR), as defined in RECIST version 1.1 criteria.

**Results:**

In this study, 15 BTC patients who met the criteria were enrolled. The ORR was 26.7%, the median progression‐free survival (mPFS) was 8.6 months (95% CI: 2.1–15.2), the median overall survival (mOS) was 14.53 months (95% CI: 0.8–28.2) and the disease control rate (DCR) was 87.6%. A patient with hilar cholangiocarcinoma was successfully converted after three cycles of treatment and underwent surgical resection. Furthermore, patient gene sequencing revealed that STK11 was mutated more frequently in patients with poor outcomes. In addition, patients with a CD8/Foxp3 ratio > 3 had a longer survival than those with a CD8/Foxp3 ratio ≤ 3 (*P* = 0.0397).

**Conclusions:**

In patients with advanced BTC, the combination of anlotinib and toripalimab demonstrated remarkable anti‐tumor potential, with increased objective response rates (ORR), longer overall survival (OS) and progression‐free survival (PFS). Moreover, STK11 and CD8/Foxp3 may be as biomarkers that can predict the effectiveness of targeted therapy in combination with immunotherapy.

## Graphical abstract

In this study, we found the combination of anlotinib (a multi‐targeted tyrosine kinase inhibitor) combined with toripalimab (a PD‐1 monoclonal antibody) demonstrated remarkable anti‐tumor potential in the treatment of unresectable biliary tract cancer (BTC). Moreover, STK11 and CD8/Foxp3 may be as biomarkers that can predict the effectiveness of targeted therapy in combination with immunotherapy.

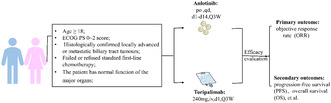

## Introduction

Biliary tract cancer (BTC) is a malignant tumor originating from the differentiation of bile ducts or gallbladder epithelial cells. It encompasses intrahepatic cholangiocarcinoma (ICC), hilar cholangiocarcinoma (HCCA), extrahepatic cholangiocarcinoma (eCCA) and gallbladder carcinoma (GBC).^1^ Radical surgery remains the mainstay of treatment for BTC, although only a limited percentage of patients (30–35%) are eligible for this approach.^2^ Moreover, the recurrence rate following surgical intervention is alarmingly high. Patients with ICC who undergo radical surgical resection experience a recurrence rate of 53.3%, with the meagre 5‐year disease‐free survival rate (DFS) of 32%.^3^

Gemcitabine combined with cisplatin is the established first‐line treatment for patients with advanced BTC. However, the median overall survival (mOS) achieved with this treatment is only 11.6–13.4 months.^4^ In the recent phase III TOPAZ‐1 study, the combination of durvalumab with gemcitabine and cisplatin demonstrated an objective response rate (ORR) of 26.7%, a mOS of 12.8 months, and a median progression‐free survival (mPFS) of 7.2 months for patients with advanced BTC.^5^ This combination has been approved by the US Food and Drug Administration (FDA) as a first‐line treatment for advanced BTC, but the trial results indicated that OS remains limited.

Limited treatment options are available for patients who progress after receiving first‐line standard chemotherapy. As a result, extensive research has been conducted to explore the potential of targeted therapy, immunotherapy and combination therapy. However, despite the adoption of the FOLFOX regimen as the standard second‐line treatment for BTC, its mOS rate of only 6.2 months remains low.^6^ Genetic sequencing of BTC has revealed that approximately 7% of cases exhibit genetic mutations, with fibroblast growth factor receptor 2 (FGFR2) mutations accounting for 6.1%.^7^ Consequently, FGFR has emerged as the primary target for targeted therapy in BTC. A phase II trial of pemigatinib, a small molecule inhibitor of FGFR1‐3, was conducted to treat 38 patients (35.5%) with FGFR2 fusion or rearrangement, resulting in complete remission in 3 patients and partial remission in 35 patients.^8^ This study led to the approval of Pemigatinib for advanced BTC. Furthermore, another FGFR inhibitor, futibatinib, has demonstrated an ORR of 41.7%, a mPFS of 9.0 months and a mOS of 21.7 months in patients with FGFR2 fusion/rearrangement in ICC.^9^

The results of tumor tissue immunohistochemistry revealed that 7.3% of ICC and 5.2% of hilar or distal cholangiocarcinoma tested positive of PD‐L1,^10^ suggesting the potential benefit of immunotherapy in BTC. However, clinical studies have not demonstrated significant efficacy of immune monotherapy for advanced BTC. For instance, the KEYNOTE‐028 study included 24 PD‐L1‐positive patients with advanced BTC who received pembrolizumab, resulting in an ORR of 13%, PFS of 1.8 months, and mOS of 5.7 months.^11^ Similarly, in the KEYNOTE‐158 study, pembrolizumab demonstrated an ORR of 5.8%, mOS of 7.4 months, and mPFS of 2.0 months in patients with advanced BTC.^11^

The efficacy of targeted monotherapy and immune monotherapy in the treatment of patients with advanced BTC is limited. However, the combination of lenvatinib and pembrolizumab has demonstrated impressive results, with an ORR of 25%, mPFS of 4.9 months (95% CI: 4.7–5.2 months) and mOS of 11.0 months (95% CI: 9.6–12.3 months) in patients with BTC of standard who have progressed after first‐line therapy.^12^ Additionally, the combination of durvalumab and tremelimumab had an ORR of 10.8% in BTC patients.^13^ These findings indicate that while targeted monotherapy and immunotherapy may have limited efficacy in treating advanced BTC, combination regimens of PD‐1 monoclonal antibody demonstrate greater therapeutic potential.

Angiogenesis is a fundamental characteristic of cancer and plays a critical role in tumor growth and progression. It has been observed that anti‐angiogenic therapy can upregulate the expression of immune checkpoints.^14^ Anlotinib, a novel multi‐target tyrosine kinase inhibitor, has demonstrated significant anti‐tumor effects. In ICC, anlotinib exerts its inhibitory effects on tumor cell proliferation and invasion by interfering with the VEGF/PI3K/AKT signalling pathway.^15^ Moreover, anlotinib has shown the potential to enhance the potency of immunotherapy by normalising the vasculature and inducing T‐cell inflammation in the tumor microenvironment (TME).^16^ Toripalimab, a humanised monoclonal antibody against programmed death protein 1 (PD‐1), blocks the interaction between PD‐1 and its ligands.^17^ Therefore, the objective of this study was to assess the safety and efficacy of anlotinib hydrochloride in combination with toripalimab for patients with unresectable BTC. Additionally, considering the complex nature of the BTC TME, we will also explore potential markers that may be associated with treatment efficacy.

## Results

### Effective

From May 2020 to September 2021, a total of 15 eligible patients were enrolled to a combination therapy of anlotinib and toripalimab. Among these patients, there were nine with ICC, three with HCCA and three with GBC. Out of the 15 patients, four (26.7%) who were unable to tolerate chemotherapy received anlotinib in combination with toripalimab as first‐line therapy, while 11 (73.3%) were undergoing second‐line therapy. The baseline characteristics of the patients are presented in Table 1.

**Table 1:** Demographic and disease characteristics at baseline

Eligible patients' characteristics	Eligible patients (*n* = 15)
Median age (IQR)	59 (38–74)
Gender, *n* (%)	
Male	9 (60.0%)
Female	6 (40.0%)
ECOG PS, *n* (%)	
0	2 (13.3%)
1	11 (73.4%)
2	2 (13.3%)
Prior line of treatment, *n* (%)	
0	4 (26.7%)
1	11 (73.3%)
Radical surgery	
Yes	9 (60%)
No	6 (40%)
Primary tumor site, *n* (%)	
Intrahepatic cholangiocarcinoma	9 (60.0%)
Hilar cholangiocarcinoma	3 (20.0%)
Gallbladder carcinoma	3 (20.0%)
Disease stage^a^	
II	2 (13.3%)
III	4 (26.7%)
IV	9 (60%)
Extrahepatic metastasis	
Lung	2 (13.3%)
Lymph nodes	12 (80%)
Bone	3 (20%)
Other	1 (6.7%)
Number of metastatic sites	
1	1 (6.7%)
≥ 2	14 (93.3%)

Data are median (IQR) or *N* (%).

ECOG, Eastern Cooperative Oncology Group.

^a^ Modified staging classification for intrahepatic cholangiocarcinoma based on the sixth and seventh editions of the American Joint Committee on Cancer and the Union for International Cancer Control TNM staging systems.

**Figure 1:**
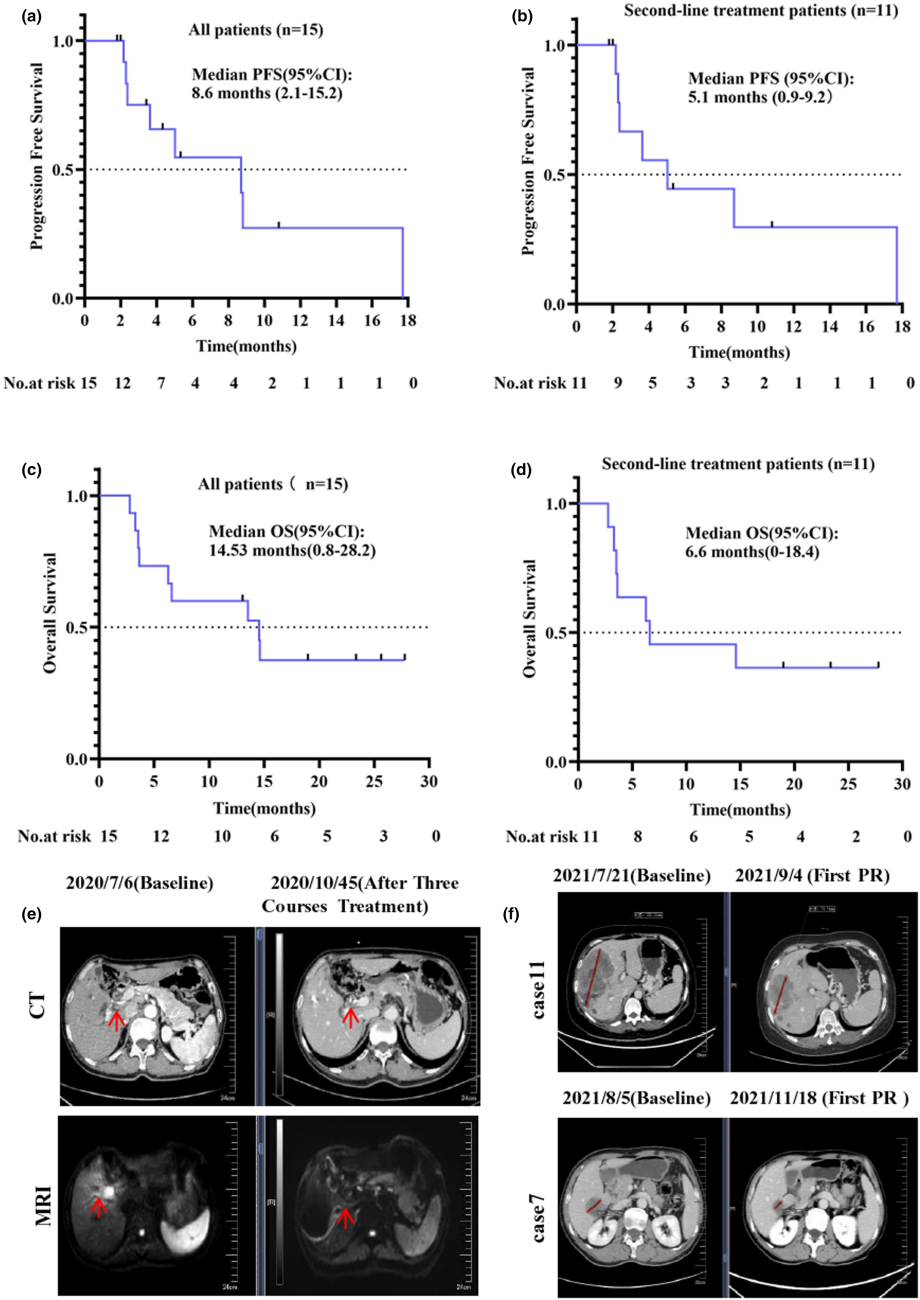
Kaplan–Meier progression‐free survival curves of all patients **(a)** and second‐line treatment patients **(b)**; Kaplan–Meier overall survival curves of all patients **(c)** and the second‐line treatment patients **(d)**. **(e)** The CT and MR of the patient with successfully converted. **(f)** The CT of two patients with the evaluated of PR.

Each patient received a minimum of two treatment cycles, and as of the data cut‐off date on 6 September 2022, all patients were evaluated. The efficacy of all patients, assessed according to RECIST v1.1, is presented in Table 2. The evaluation demonstrated a PR in 4 patients, SD in 9 patients, PD in two patients. The ORR was 26.7% (4/15), and the DCR was 86.7% (13/15). The mPFS was 8.6 months (95% CI: 2.1–15.2) for all patients (Figure 1a) and 5.1 months (95% CI: 0.9–9.2) for patients who received second‐line treatment (Figure 1b). The mOS was 14.53 months (95% CI: 0.8–28.2) for all patients (Figure 1c) and 6.6 months (95% CI: 0–18.2) for patients who received second‐line treatment (Figure 1d). Two patients achieved PR after 2 cycles of treatment, and the corresponding computed tomography images are presented in Figure 1f.

**Table 2:** Best overall response and disease control rate

Response % (*n*)	First‐line treatment eligible patients (*n* = 4)	Second‐line treatment eligible patients (*n* = 11)
CR	0	0
PR	50.0% (2)	18.2% (2)
SD	50.0% (2)	63.6% (7)
PD	0	18.2% (2)
ORR	50.0% (2)	18.2% (2)
**ORR‐Total**	**26.7% (4)**	
DCR	100.0% (4)	81.8% (9)
**DCR‐Total**	**86.7% (13)**	

Furthermore, one patient (Patient No. 2) in this study demonstrated successful conversion following treatment with the proposed protocol. This patient, a 68‐year‐old man diagnosed with HCCA in stage IIIB in July 2020, underwent three cycles of anlotinib and toripalimab treatment. The imaging evaluation of this patient is presented in Figure 1e. Following a multidisciplinary treatment (MDT) discussion, it was determined that the patient was eligible for radical surgical intervention. On 1 December 2020, the patient underwent surgical treatment. Subsequently, he received oral tegafur for 6 months, resulting in tumor‐free survival.

### Safety

The majority of patients (14 out of 15, 93.3%) experienced adverse reactions, which were predominantly mild in severity (Grades 1 and 2). Notably, no Grade 4 adverse reactions were observed. As presented in Table 3, these adverse reactions included thrombocytopaenia (60.0%), leucopoenia (53.3%), hypertension (20.0%), malaise (13.3%), hypothyroidism (6.7%) and fever (6.7%). In addition, Grade 3 adverse reactions observed were hypertension and reduced platelet count.

**Table 3:** Patients with treatment‐related AEs

TRAEs	Grade 1–2% (*n*)	Grade 3% (*n*)	Grade 4% (*n*)
Platelet count decreased	60.0% (9)	0	0
Leukocyte count decreased	53.3% (8)	0	0
Hypertension	20.0% (3)	0	0
Fatigue	13.3% (2)	13.3% (2)	0
Fever	6.7% (1)	0	0
Hypothyroidism	6.7% (1)	0	0

### Results of patients NGS assessment

**Figure 2:**
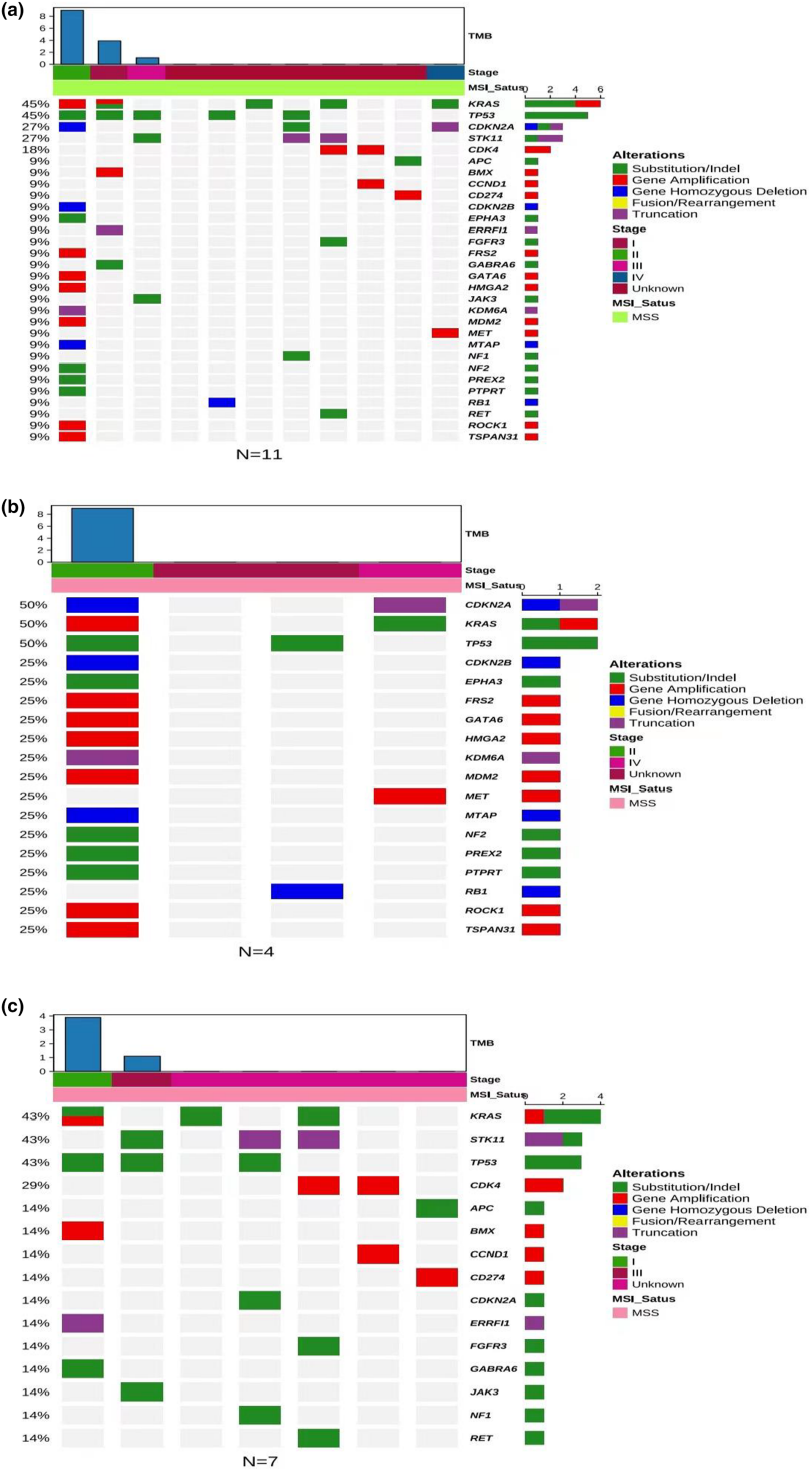
Next‐generation sequencing (NGS) gene sequencing results. **(a)** Mutations in overall patients. **(b)** Efficacy evaluation for PR patients with mutations. **(c)** Efficacy evaluation for SD and PD patients with mutations.

Tumor tissues from 11 patients in this study were collected for next‐generation sequencing (NGS) analysis. The identified mutated genes were primarily TP53, KRAS, CDKN2A, STK11 and CDK4 (Figure 2a). Additionally, a comparative analysis was conducted between patients with a favourable efficacy evaluation (PR) as the good efficacy group (*N* = 4) and those with an unfavourable efficacy evaluation (PD and SD) as the poor efficacy group (*N* = 7). TP53, KRAS and CDKN2A were commonly gene mutated in the good group (Figure 2b). In contrast, TP53, KRAS and STK11 were commonly mutated genes in the poor efficacy group (Figure 2c). Notably, the *STK11* gene exhibited a mutation frequency of 43% in the poor efficacy group, whereas no mutations in this gene were identified in the good efficacy group.

### Tumor markers expression analysis by immunohistochemistry (IHC)

**Figure 3:**
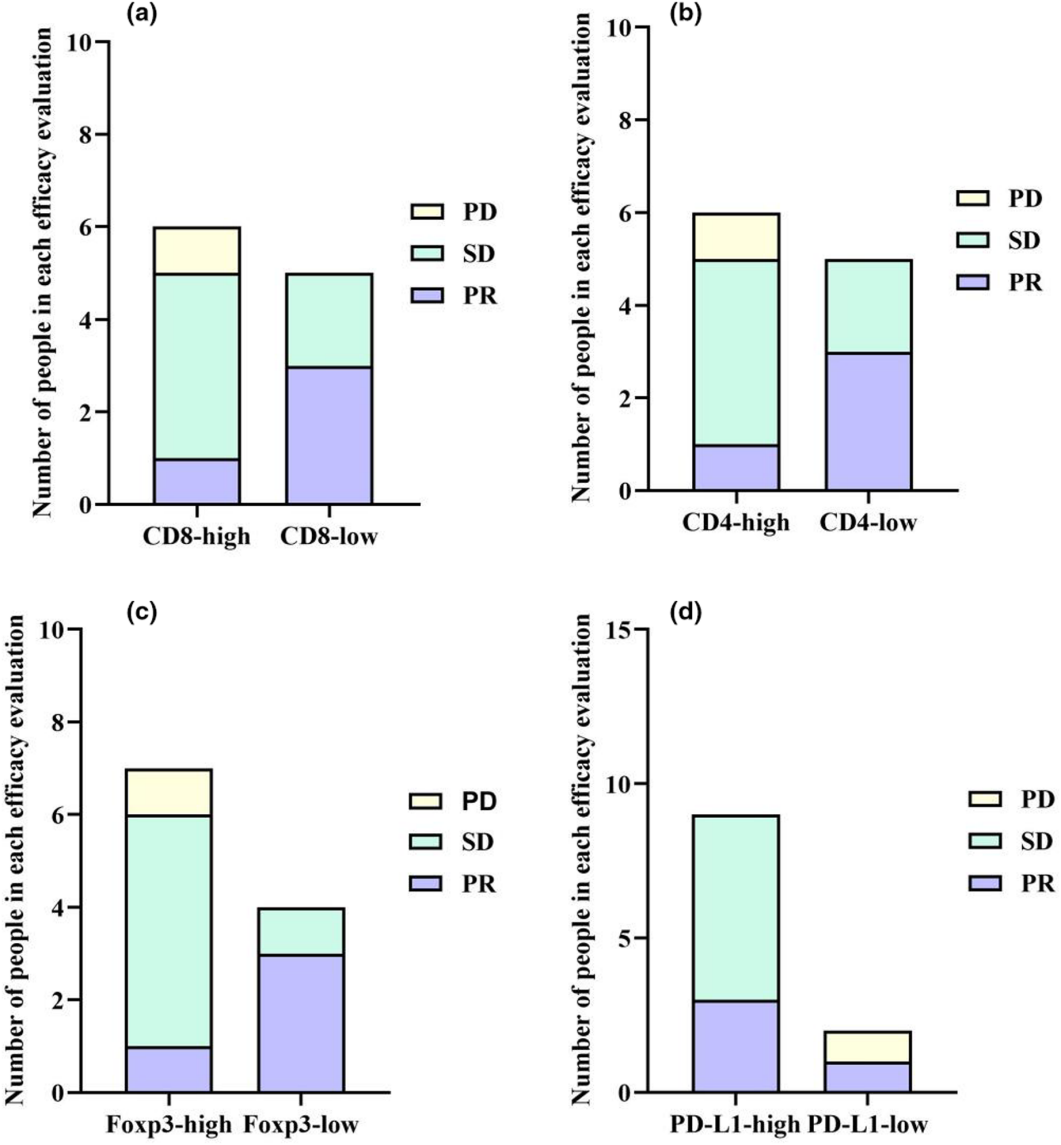
Number of people in each efficacy evaluation among the different expressed tumor markers.

**Figure 4:**
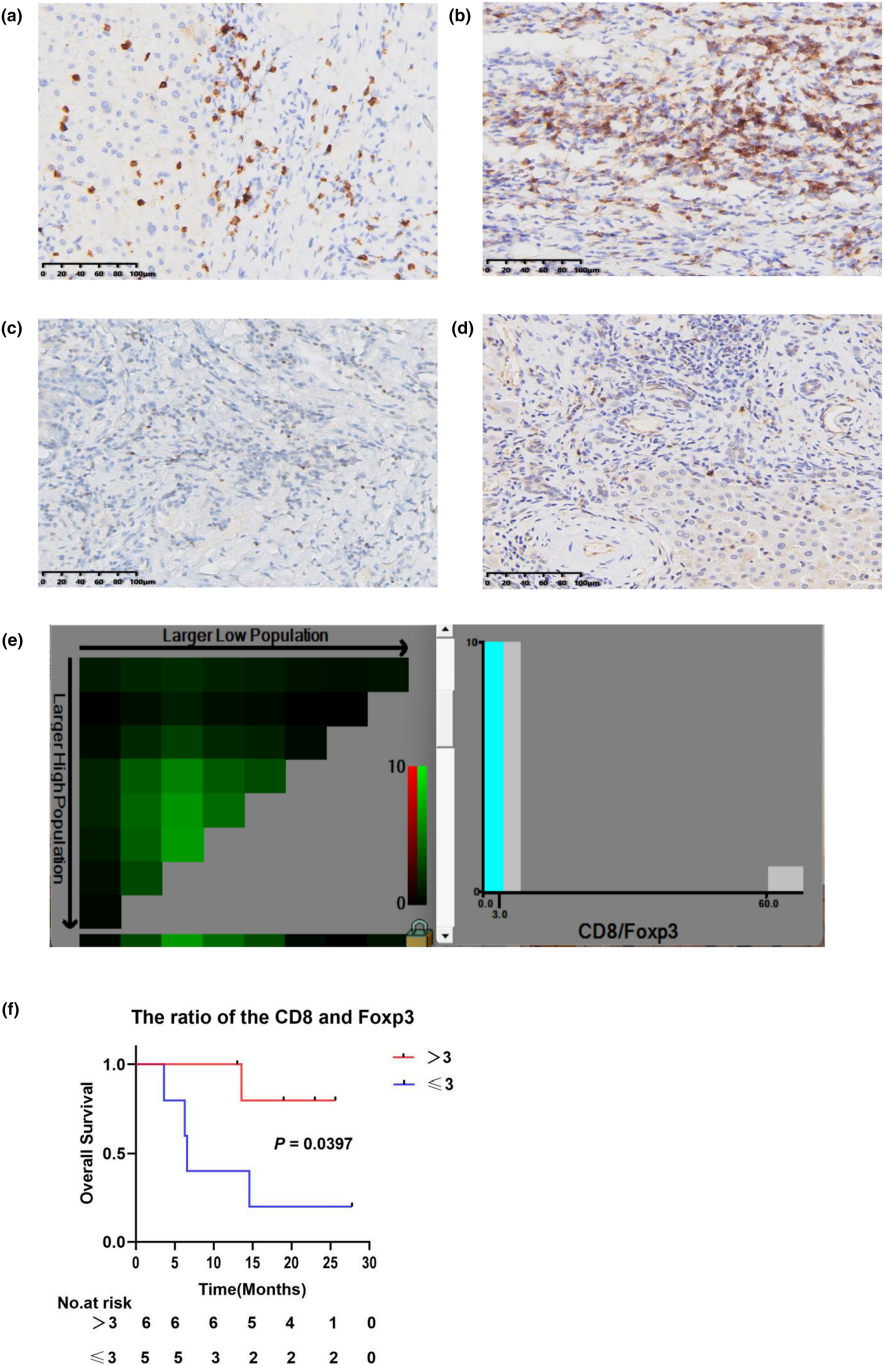
Immunohistochemical straining image of CD8 **(a)**, CD4 **(b)**, Foxp3 **(c)** and PD‐L1 **(d)** of a patient with successfully converted **(e)**. X‐tile analysis of the CD8/Foxp3. The optimal cut‐off value of CD8/Foxp3 is 3.0 **(f)**. Kaplan–Meier overall survival curves of the CD8/Foxp3.

To further characterise the molecular profile of patients who responded to the treatment, immunohistochemistry (IHC) analysis was performed to examine the expression of CD8, CD4, Foxp3 and PD‐L1 in 11 patients with efficacy evaluations including PR, SD and PD. The expression levels of these biomarkers in all patients are shown in Supplementary table 2. Figure 3 illustrates that patients with PR showed a higher prevalence of low expression of Foxp3 and high expression of PD‐L1. To consolidate these findings in terms of OS, we established the parameter CD8/Foxp3. X‐tile analysis the optimal cut‐off values of CD8/Foxp3 ratio is 3.0 (Figure 4e). Patients in the CD8/Foxp3 ratio > 3 group exhibited prolonged survival compared with those in the CD8/Foxp3 ratio ≤ 3 group (*P* = 0.0397) (Figure 4f). As mentioned earlier, patient NO.2 was a successfully converted patient. Analysis of their immunohistochemical background revealed significant expression of CD8 (Figure 4a), CD4 (Figure 4b), Foxp3 (Figure 4c) and PD‐L1 (Figure 4d).

## Discussion

Biliary tract cancer constitutes approximately 3% of all gastrointestinal malignancies and ranks as the second most prevalent hepatobiliary cancer.^18^ Because of its high malignancy and aggressive nature, effective systemic treatment options for advanced BTC are limited. Currently, chemotherapy stands as the mainstay treatment for advanced BTC. However, there exist limited data to assess the potential advantages of combining targeted therapy with immunotherapy in the management of BTC. This phase II clinical trial aimed to evaluate the safety and efficacy of anlotinib in combination with toripalimab was assessed in patients with advanced BTC.

In this study, the first‐line treatment achieved an ORR of 50% and a DCR of 100%. In the second‐line treatment, the ORR was 18.2% and the DCR was 81.8%. The majority of observed adverse events were Grade 1 or 2, with no reports of serious immune‐related adverse events. Notably, one patient with ICC achieved successful conversion after receiving three cycles of anlotinib in combination with toripalimab as first‐line treatment. These findings highlight the potential of targeted combination immunotherapy as a viable treatment approach for advanced BTC.

LKB1(STK11) is an oncogene implicated in the proliferation and migration of cancer cells.^19^ The downregulation of LKB1 has been found to significantly enhance the Wnt/β‐catenin signalling pathway in ICC cells.^20^ Additionally, studies have reported that silencing LKB1 can lead to upregulation of PD‐L1 surface expression in ICC cells.^21^ Our study's gene sequencing analysis revealed a higher prevalence of STK11 (LKB1) mutations in patients with unfavourable outcomes. This suggests that individuals with a high frequency of SKT11 mutations may exhibit lower PD‐L1 expression, which could potentially compromise the effectiveness of their treatment.

The efficacy of immunotherapy is often influenced by the interactions between tumor cells and the TME.^22^ CD8, CD4 and Foxp3 are markers of T cells, which are important components of the TME.^23^, ^24^ Despite the high PD‐L1 expression of PD‐L1, tumor‐infiltrating lymphocytes (TILs) and tumor mutational burden (TMB) in BTC, the response to immunotherapy has been reported to be unsatisfactory.^11^, ^25^, ^26^ Our analysis of these markers yielded similar results, showing no correlation between the expression of CD8, CD4, PD‐L1 and Foxp3 and the efficacy of immunotherapy, although the sample size was limited. However, a multicentre randomised phase II trial of atezolizumab with or without cobimetinib for BTC revealed increased expression of antigen processing and presentation gene and higher CD8/FoxP3 ratios following combination therapy, indicating a potential correlation between the CD8/Foxp3 ratio and patient overall survival.^27^ Therefore, the findings of this study suggest that the CD8/Foxp3 ratio may serve as a critical factor in determining patient survival.

This phase II study investigating the combination of anlotinib and toripalimab in patients with BTC has shown promising results. Nevertheless, it is important to note that the study had limitations in terms of sample size and the heterogeneity of the patient population. Hence, further research is warranted to validate these findings, employing larger sample sizes and focusing on more refined tumor subtypes. In conclusion, this study suggests that combination therapy could represent a novel and effective treatment approach for advanced BTC. Moreover, certain markers such as STK11 and the CD8/Foxp3 ratio may indicate potential benefit from this treatment.

## Methods

### Experimental procedure

#### Study design and patients

This is a single‐centre, prospective, single‐arm, exploratory clinical study to assess the safety and efficacy of anlotinib hydrochloride in combination with toripalimab for the treatment of unresectable BTC.

Patient inclusion criteria were as follows: (1) Age ≥ 18 years, patients volunteered to participate in the study and signed an informed consent form (ICF). (2) Histologically confirmed locally advanced or metastatic biliary tract tumors (including ICC, HCCA, eCCA and GBC). (3) Patients who failed first‐line chemotherapy or who chose not to undergo first‐line chemotherapy. (4) Disease progression within 14 months prior to enrolment (RECIST1.1 criteria must be used as the basis for assessing disease progression). (5) Patients who had normal function of major organs. (6) ECOG PS: 0–2 score. (7) Expected survival ≥ 3 months.

Exclusion criteria were as follows: (1) Recent use (within 6 months) of VEGFR‐TKI small molecule drugs. (2) Patients who had been treated with any antibodies/drugs targeting T‐cell co‐regulatory proteins (immune checkpoints). (3) Confirmed allergy to the investigational drug and/or its excipients. (4) Patients who could not take oral medication. (5) People with high blood pressure that could not be reduced to within the normal range with antihypertensive medication. Detailed study criteria can be found in Supplementary table 1.

#### Procedures

All patients enrolled were treated with anlotinib hydrochloride in combination with toripalimab. For the treatment regimen, patients were administered Anlotinib Hydrochloride Capsules at a dose of 12 mg orally once daily before breakfast for 2 weeks, followed by a 1‐week break period, making up a 21‐day cycle. Toripalimab was given intravenously at a dose of 240 mg on the first day, also for a 21‐day cycle. Patients achieving complete remission (CR), partial remission (PR) and stable disease (SD) in response to the treatment continued to receive medication until disease progression, intolerable toxicity or patient request to discontinue the treatment. If drug toxicity caused a suspension of treatment, the cumulative duration of suspension should not exceed 2 weeks per dosing cycle, and no more than two pauses per cycle to ensure the proper intensity of treatment. The trial will be terminated for patients whose treatment cycle is delayed by more than 2 weeks, as well as those who exceed the above criteria.

#### Efficacy evaluation and statistical analysis

Primary study endpoints included the objective response rate (ORR), which was assessed every 6 weeks to determine the efficacy. Secondary study endpoints included progression‐free survival (PFS), overall survival (OS) and disease control rate (DCR). The efficacy was assessed based on the RECIST version 1.1 criteria. Point estimates and 95% confidence intervals were provided for efficacy endpoints such as ORR and DCR, and intervals were estimated using the Clopper‐Pearson method. The Kaplan–Meier method was utilised to estimate PFS and OS and to calculate 95% confidence intervals.

#### Gene sequencing

Paraffin sections from 11 patients involved in this clinical trial for NGS (next‐generation sequencing) analysis. NGS analysis was performed at a CLIA/CAP‐compliant Molecular Diagnostics Service laboratory of OrigiMed Co., Ltd (Shanghai, China). The 706 tumor‐related gene panel sequencing method was used. To begin, at least 50 ng DNA was extracted from each 40 mM formalin‐fixed paraffin‐embedded (FFPE) tumor sample using a DNA extraction kit (QIAamp DNA FFPE Tissue Kit). Subsequently, genes were captured and sequenced using the Illumina NextSeq 500 platform (Illumina Incorporated, San Diego, CA, USA).

#### Immunohistochemistry

The expression of CD4, CD8, Foxp3 and PD‐L1 was evaluated in pathological specimens of 11 patients at our institution. The tissue sections were stained following the steps of section preparation, antigen repair, antibody hybridisation, colour development and sealing. The results were interpreted by two experienced pathologists in a double‐blind method. Tumor cells and lymphocytes expressing PD‐L1 were considered positive, while CD4 and CD8 with a value of ≥ 10 were considered to be high expression. A Foxp3 value greater than 1 was also considered to be high expression.

## Ethical approval

This study protocol was reviewed and approved by ethics committee of Comprehensive Cancer Center of Drum Tower Hospital of Nanjing University and Drum Tower Hospital of Nanjing University. The patients/participants provided their written informed consent to participate in this study.

## Author contributions

**Mingzhen Zhou:** Data curation; formal analysis; writing – original draft. **Yuncheng Jin:** Data curation; investigation. **Sihui Zhu:** Data curation; investigation. **Chen Xu:** Data curation; investigation. **Lin Li:** Data curation; investigation. **Baorui Liu:** Conceptualization; resources; supervision; writing – review and editing. **Jie Shen:** Conceptualization; funding acquisition; resources; supervision; writing – original draft; writing – review and editing.

## Conflict of interest

The authors declare that they have no conflict of interests.

## Funding information

The study was supported by the National Natural Science Foundation of Nanjing University of Chinese Medicine (No. XZR2023075); the Hospital Management Research of Jiangsu Province (No. JSYGY‐3‐2023‐618); and the Medical Science and Technology Development Foundation of Nanjing (No. YKK22095).

## Consent for publication

The patients/participants provided their written informed consent to participate in this study.

## Supporting information


Supplementary table 1
Supplementary table 2

## Data Availability

All data generated or analysed during this study are included in this published article.
