# Supplementary material for: A phase II study to evaluate the safety and efficacy of anlotinib combined with toripalimab for advanced biliary tract cancer
Source: Clin Transl Immunology. 2024 Jan 12;13(1):e1483. doi: 10.1002/cti2.1483 (PMC10786709; doi:10.1002/cti2.1483)
Supplement: Supplementary file 1 — Supplementary table 1 Supplementary table 2 [file CTI2-13-e1483-s001.docx]

**Supplementary table 1.** Patient inclusion and exclusion criteria

| Inclusion criteria | 1.Age ≥ 18 years |
| --- | --- |
|  | 2. Histologically confirmed locally advanced or metastatic biliary tumours (including intrahepatic cholangiocarcinoma, Hilar cholangiocarcinoma, extrahepatic cholangiocarcinoma and Gallbladder carcinoma). |
|  | 3.Patients who have progressed through first-line chemotherapy, or who do not wish to receive first-line chemotherapy. |
|  | 4. Disease progression within 14 months prior to enrolment (RECIST criteria must be used to assess disease progression). |
|  | 5.Normal function of the major organs in accordance with the following requirements:  (a) HB≥90 g L^-1^  (b) ANC ≥1.5×10^9^ L^-1^  (c) PLT ≥80×10^9^ L^-1^  (d) BIL <1.5 times the upper limit of normal (ULN)  (e) ALT and AST <2.5 × ULN; in the case of liver metastases, ALT and AST <5 × ULN  (f) Serum Cr ≤ 1 × ULN and endogenous creatinine clearance ≥ 50 ml min^-1^ (Cockcroft-Gault formula). |
|  | 6. ECOG PS: 0-2 score. |
|  | 7. Expected survival ≥ 3 months |
|  | 8. Good cardiac function before admission, no myocardial infarction within six months, hypertension, other coronary artery disease currently under control within. |
|  | 9. No other uncontrollable benign disease such as lung, kidney or liver infection prior to enrollment. |
|  | 10. Women of childbearing potential must have a negative pregnancy test (serum or urine) within 7 days prior to enrolment and voluntarily use an appropriate method of contraception during the observation period and for 6 months after the last dose. For men, surgical sterilization or agreement to use an appropriate method of contraception during the observation period and for 6 months after the last dose. |
|  | 11. Patients voluntarily enrolled in this study and signed an informed consent form (ICF) |
|  | 12. Good compliance is expected with the ability to follow up on efficacy and adverse effects as required by the protocol. |

| Exclusion criteria | 1.Patients who have been treated with VEGFR-TKI small molecule drugs; or patients who have been treated with any antibody/drug targeting T-cell co-regulatory proteins (immune checkpoints) including PD-1, PD-L1, CTLA4, TIM3 and LAG3 |
| --- | --- |
|  | 2. Patients with proven hypersensitivity to the investigated drug and/or its excipients |
|  | 3. Patients have multiple factors that affect the absorption of oral medications (e.g. inability to swallow, nausea and vomiting, chronic diarrhoea and intestinal obstruction). |
|  | 4. People with high blood pressure that cannot be reduced to the normal range with antihypertensive medication. |
|  | 5. Patients with other serious medical conditions that are difficult to control (including atrial fibrillation, angina pectoris, cardiac insufficiency, ejection fraction below 50%, renal insufficiency with a urine protein test of 2+ or more). |
|  | 6. Patients with a risk of gastrointestinal bleeding should not be enrolled, including the following conditions: a. patients with active digestive ulcer disease and fecal occult blood (+ +); b. patients with a history of melena and hematemesis within 3 months. |
|  | 7. Patients at risk of gastrointestinal bleeding cannot be enrolled. Abnormal coagulation (INR > 1.5× ULN, APTT > 1.5× ULN), with bleeding tendency. |
|  | 8. Pregnant or breastfeeding women. |
|  | 9. Patients have received live vaccination within 28 days prior to treatment. |
|  | 10. Patients with active, or previous, autoimmune diseases that are at risk of recurrence (e.g. systemic lupus erythematosus, rheumatoid arthritis, inflammatory bowel disease, autoimmune thyroid disease, vasculitis, psoriasis) or are at risk of such (e.g. Having received an organ transplant requiring immunosuppressive treatment). However, subjects were further screened for type I diabetes, hypothyroidism with only hormone replacement therapy, skin disease without systemic treatment (e. g., vitiligo, psoriasis, or alopecia), or not expected to recur in the absence of external triggers. |
|  | 11. Patients were diagnosed of immunodeficiency disease or who have received systemic hormone therapy within 14 days prior to treatment (e.g. equivalent to >10 mg per day) or have used any other form of immunosuppressive therapy. Note: If the subject does not have an active Hormones equivalent to ≤10 mg prednisone per day are permitted as adrenal replacement therapy if the subject does not have an active autoimmune disease. Subjects are permitted to Topical, ophthalmic, intra-articular, intranasal and inhaled glucocorticoids are permitted (minimal systemic absorption). The use of short duration Glucocorticoids are permitted for prophylaxis (e.g. for contrast allergy) or for the treatment of non-autoimmune diseases (e.g. contrast allergy-induced delayed hypersensitivity reactions). |
|  | 12. Subjects with active chronic hepatitis B or active hepatitis C. Subjects who are positive for hepatitis B virus surface antigen (HBsAg) or hepatitis C virus (HCV) antibodies during the screening period must be further tested by quantitative hepatitis B virus (HBV) DNA testing (excluding those above 2500 copies [cps] mL^-1^ or 500 IU mL^-1^) and HCV RNA testing (excluding those(exceeding the lower limit of detection of the assay), after exclusion of active hepatitis B or C infection requiring treatment. Hepatitis B carriers, medically stable hepatitis B (DNA titer must not be higher than 2500 copies [cps] mL^-1^ or 500 IU mL^-1^) and cured hepatitis C subjects may be enrolled. |
|  | 13. Active infection requiring systemic treatment 14 days prior to treatment. |
|  | 14. Patients with a known history of human immunodeficiency virus (HIV) virus infection and/or acquired immunodeficiency syndrome. |
|  | 15. Those deemed unsuitable for inclusion by the researcher |

**Supplementary table 2.** The expressions of CD8, CD4, Foxp3 and PD-L1 in 11 patients

| **Biomarker** | **High Expression / Positive** | | | **Low Expression / Negative** | | |
| --- | --- | --- | --- | --- | --- | --- |
|  | PR | SD | PD | PR | SD | PD |
| **CD8** | 1 | 4 | 1 | 3 | 2 | 0 |
| **CD4** | 1 | 4 | 1 | 3 | 2 | 0 |
| **Foxp3** | 1 | 5 | 1 | 3 | 1 | 0 |
| **PD-L1** | 3 | 6 | 0 | 1 | 0 | 1 |
